# Decision Support Tools in Deceased Donor Kidney Transplantation: An Environmental Scan and Appraisal of Online Resources

**DOI:** 10.1097/TP.0000000000005473

**Published:** 2025-07-14

**Authors:** Sarah L. White, Rachel B. Cutting, Matilda McLean, Danielle M. Muscat, Pinika Patel, Angela C. Webster

**Affiliations:** 1 Sydney School of Public Health, Faculty of Medicine and Health, University of Sydney, Camperdown, NSW, Australia.; 2 Sydney Health Literacy Lab, Sydney School of Public Health, Faculty of Medicine and Health, University of Sydney, Camperdown, NSW, Australia.; 3 Psycho-Oncology Co-operative Research Group, School of Psychology, Faculty of Science, University of Sydney, Camperdown, NSW, Australia.; 4 National Health and Medical Research Council Clinical Trials Centre, University of Sydney, Camperdown, NSW, Australia.; 5 Westmead Applied Research Centre, Westmead Hospital, Westmead, NSW, Australia.

## Abstract

The pathway to deceased donor kidney transplantation involves complex risk assessment and decision-making by donor coordinators, transplant clinicians, and patients. We reviewed freely available tools designed to facilitate decision-making in the context of deceased donor kidney transplantation for usability and communication efficacy. An environmental scan was conducted using Google; search results meeting inclusion criteria were appraised by 3 independent reviewers using the International Patient Decision Aids Standards Instrument, the Patient Education Material Evaluation Tool, and the Healthcare Systems Usability Scale. In total, 3885 search results were reviewed, from which 6 patient-directed decision aids, 1 clinician-directed decision aid, and 10 risk calculators were identified. Of the 6 patient-directed decision aids, 3 met International Patient Decision Aids Standards Instrument qualification criteria; none met certification criteria. Four of 6 decision aids were understandable by Patient Education Material Evaluation Tool criteria, whereas only 1 was actionable. Mean Healthcare Systems Usability Scale scores for the 10 risk calculators identified ranged from 50% (indicating major usability concerns) to 86% (indicating good usability with potential for improvements), with a median score of 71%. More high-quality decision support tools are needed for use in deceased donor kidney transplantation, given the complexity of decisions involved, the recognized importance of effective knowledge transfer, the value of individualized prognostic information, and the benefits of structured approaches to shared decision-making in this context.

## Graphical abstract

**Figure:**
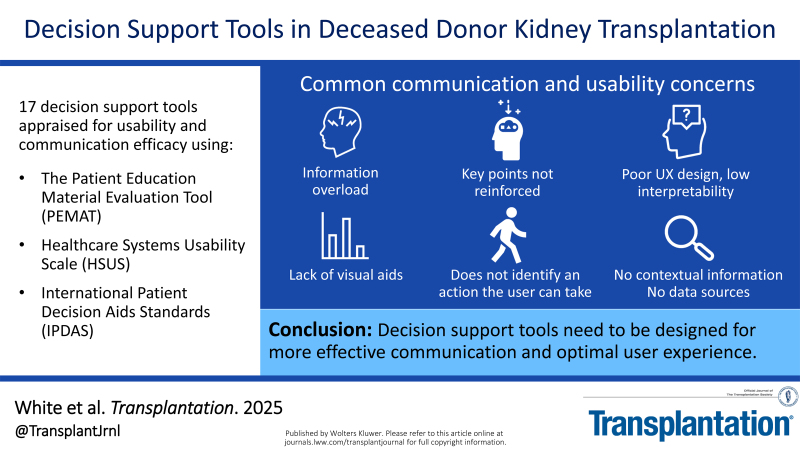

## INTRODUCTION

When considering whether a potential deceased donor should proceed to donation, an appraisal of medical suitability is made based on multiple factors, including age of the potential donor, cause of death, comorbidities, history of cancer, and presence of infectious diseases. For the potential kidney recipient and their treating clinician, a decision must then be made whether to accept the risks associated with a given donor or to instead choose the associated risks—and the impact on quality of life—of staying on dialysis. Donor coordinators, transplant clinicians, and patients are each required to make complex and consequential decisions along the pathway from potential donor assessment to organ retrieval and subsequent kidney transplantation.

For clinicians and donor coordinators, local clinical guidelines may provide advice on the level of risk associated with potential donor characteristics; however, correctly interpreting and acting on such advice in real-world, time-sensitive donation scenarios can be challenging.^1^ Combining information on kidney quality, history of malignancy, infectious disease risk, as well as biological factors such as donor size and ischemia time, then weighing donor-related risks against the needs and preferences of a given recipient, is a complex task that is made more difficult by often incomplete donor histories and uncertainty around risk estimation. This complexity may result in risk-averse decisions and therefore lost opportunities for donation and transplantation.

For patients, informed participation in shared decision-making regarding whether to accept a given kidney requires (1) effective communication of the risks and benefits of transplantation versus dialysis, (2) incorporation of patient views, and (3) structured approaches to decision-making that enable a consensus to be reached that is aligned with patient values and preferences.^2^ Risk communication is a critical component of shared decision-making, as patients need to be able to conceptualize the relative risks and benefits of available treatment options to choose between them.^2^ Previous research into the decision support needs of patients on the kidney transplant waiting list indicates that a lack of awareness regarding likely waiting list outcomes and individual prognosis are key barriers to informed decision-making about donor offers.^3^

Decision support tools such as risk calculators and decision aids may help donor coordinators, clinicians, and patients to make more confident, evidence-based decisions at critical points in the donation and transplantation pathway (Table 1). Risk calculators can synthesize information on donor-related risk factors to generate evidence-based risk estimates that may reduce uncertainty around donor medical suitability decisions. They can also incorporate recipient information to generate individually tailored prognostic estimates, which can be used to increase patient understanding of the risks and benefits of accepting a given kidney offer.^4^ By comparison, patient decision aids are “structured interventions designed to help people make specific and deliberative choices among options” by assimilating evidence about the decision context, patient values and preferences, the relative risks and benefits of treatment options, and likely outcomes.^1,5^ People who are exposed to patient decision aids have been found to have more accurate risk perception, greater congruence between values and choices, and reduced decisional conflict.^6^

**TABLE 1.** Decision points on the transplantation pathway for patients choosing kidney transplantation and for transplant nephrologists

Perspective	Decision point
Clinicians	• Is my patient ready for kidney transplantation? • Is this donor medically suitable for kidney donation? • Does the extent of the infectious disease/cancer risk associated with this donor preclude acceptance for my patient? • Does this kidney offer a sufficient benefit to my patient? (ie, is the quality/expected longevity of this kidney sufficient for the intended recipient?) • Can my patient afford to wait for a better offer?
Patients	• Am I ready for kidney transplantation? • Should I pursue living or deceased donor kidney transplantation? • Which transplant center should I list at? (applicable in some countries) • Should I consent to receiving offers of kidneys where there is a degree of biovigilance risk? • Should I consent to receive offers of marginal/expanded criteria donor kidneys • Should I accept or decline this kidney offer? (ie, will I be better or worse off? How long would I wait for the next offer?)

The aim of this review was to conduct an environmental scan of freely available online risk calculators and decision aids designed to assist patients, donor coordinators, and clinicians with decisions along the pathway to deceased donor kidney transplantation. We focused on decision support tools designed to support decision-making at the point of being waitlisted; decision aids related to upstream decisions, such as whether to pursue transplantation, remain on dialysis, or seek supportive care, were excluded from this review. We evaluated the communication efficacy of identified resources using validated usability scales^7^ and health literacy guidelines.^8^

## MATERIALS AND METHODS

### Environmental Scan

Environmental scans are an established methodology in health services research with the advantage of being able to account for various types of information from diverse sources.^9,10^ Given the likelihood that many decision support tools would not have been the subject of peer-reviewed publications, an environmental scan using the Google search engine was identified as the appropriate search methodology for this study.

English language search terms were entered into the Google search engine as per Table 2. The first 100 results, excluding advertisements, were exported using a browser plugin, SEOQuake. The browser cache was cleared before each of the 40 searches. This search strategy was informed by similar previous studies.^10,11^

**TABLE 2.** Search terms used for Google search

Box 1	Box 2
Decision aid Decision support Decision tool	Kidney donation Kidney transplant Increased-risk kidney donor Deceased kidney donor
Risk assessment Risk calculator Risk profiler Risk score Risk index Risk test Risk prediction	

Phrases in Box 1 were combined with those in Box 2, using the word “and” to create 40 unique search terms.

A preliminary scoping search was conducted in March 2022 by coauthor P.P. This search was then repeated in August 2023 by an independent reviewer (R.B.C.). Searches were conducted from Sydney, Australia. Results from both searches were screened by the 2 lead researchers (S.L.W. and R.B.C.) against inclusion criteria.

### Screening of Results

Resources were included in the appraisal if they met the following criteria:

1. Presented a risk calculator or decision aid
2. Focused on deceased kidney donation and/or transplantation
3. Targeted clinicians or patients
4. Were freely accessible
5. Were available in English.

Resources were excluded if they met the following criteria:

1. Pay-to-access material
2. Focused on organs other than kidneys
3. Focused on living donation
4. Focused on earlier stages of chronic kidney disease (eg, time-to-kidney-failure risk calculators)
5. Focused on advanced care planning or supportive care for end-stage kidney disease
6. Focused on decision-making related to choice of dialysis modality or transplantation versus dialysis, as opposed to decision-making once a patient is waitlisted
7. Purely educational resources
8. Research papers or textbook excerpts that did not present a decision support tool (eg, papers on risk estimation or validation of risk estimation equations)
9. Pathologic scoring systems and diagnostic clinical decision support systems
10. Other resources not about kidney donation or transplantation.

For those resources meeting the inclusion criteria, the content was rated by 3 independent raters, and major differences were resolved by consensus discussions.

Three independent reviewers (M.M., R.B.C., and S.L.W.) evaluated patient decision aids against the International Patient Decision Aids Standards instrument (IPDASi 4.0).^12^ The International Patient Decision Aids (IPDAS) checklist was developed to establish quality standards for the certification of patient decision aids.^13^ Version 4.0 of this checklist contains 44 items across 3 categories: qualifying criteria (required in order for an intervention to be considered a decision aid), certification criteria (without which a decision aid is judged to have a high risk of harmful bias), and quality criteria.^12^ Criteria used for defining a patient decision aid were rated on a yes or no scale and certification criteria were rated on a 4-point Likert scale (1 = strongly disagree to 4 = strongly agree). To be defined as a patient decision aid, all 6 qualifying criteria must be met; to reach certification criteria, resources must score ≥3 on all 6 certification criteria.^12^ Quality criteria were assessed only for those tools that met the IPDAS qualifying criteria; the criteria were rated on a 4-point Likert scale (1 = strongly disagree to 4 = strongly agree).

Patient-directed decision aids were then appraised by 3 independent reviewers (M.M., R.B.C., and S.L.W.) using the Patient Education Material Evaluation Tool (PEMAT).^14^ Items in the PEMAT are divided into 2 domains: (1) understandability, a measure of how well the reader is able to process and explain the key message, and (2) actionability, a measure of how well a person is able to identify what to do based on the information presented. Items are rated on a binary yes or no scale, nonrelevant items can be skipped, and the final PEMAT score is the sum of scores in the understandability and actionability domains, divided by the number of applicable items. Scores from each independent reviewer were averaged to give the final PEMAT score, expressed as a percentage. A threshold of 70% was used as the indicator of whether a decision aid was understandable or actionable.^10^ Interrater reliability was assessed by calculating the intraclass correlation coefficient (using a 2-way random-effects model and absolute agreement as the relationship among raters)^15^ for understandability and actionability scores.

Online risk calculators were appraised using an adapted version of the Healthcare Systems Usability Scale (HSUS), a recently published scale for assessing the ease of use of computer-based clinical decision support systems.^7^ The authors recommend tailoring the HSUS to each application and omitting items in the scale that are not applicable to a given decision support system; therefore, a subset of the full list of HSUS items was used for the purposes of this appraisal (**Table S1, SDC,** https://links.lww.com/TP/D287). Each item was rated on a 5-point Likert scale, and the total score across all items was converted into a percentage score. Percentage scores from each independent reviewer were averaged to give the final usability score. A usability score between 20% and 50% indicated a critical need to address the usability issues of the tool; between 50% and 70% indicated a need to address the usability concerns of the tool, some of which may be major; between 70% and 90% indicated a good usability score with the potential to improve; and between 90% and 100% indicated an excellent and easy-to-use tool.^7^ Interrater reliability was assessed by calculating the intraclass correlation coefficient across HSUS scores.^15^

## RESULTS

### Search Results

A total of 3885 search results were reviewed. After the eligibility screening process, we identified 40 resources for a full appraisal, of which 17 were included in the final evaluation. These comprised the following:

1. Seven decision aids: 6 designed to support informed decision-making by patients choosing to be waitlisted for kidney transplantation and one targeted at clinicians.
2. Ten risk calculators assessing donor quality, recipient prognosis, or forecasting the outcomes of decisions related to deceased donor kidney transplantation.

**FIGURE 1.**
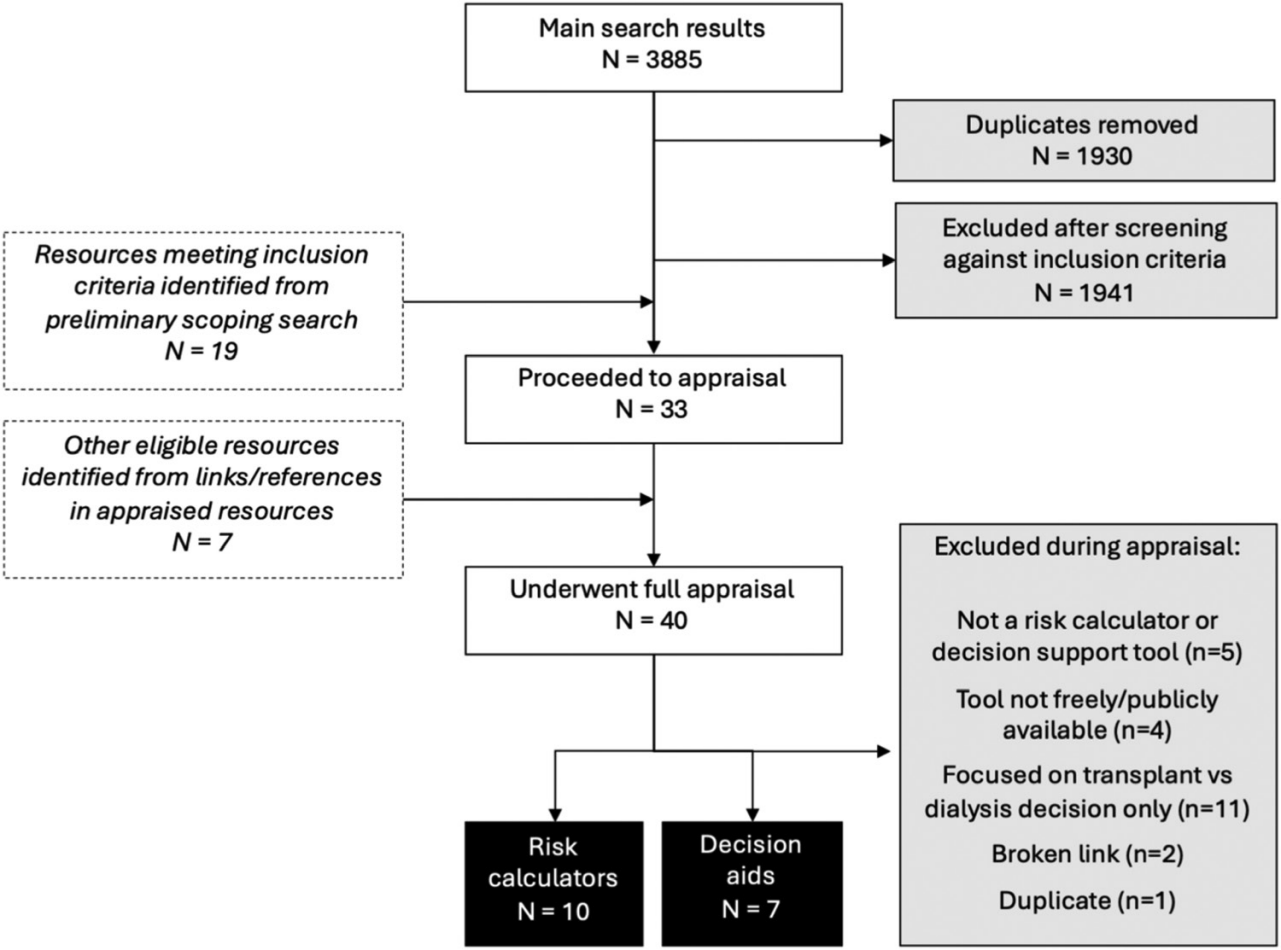
Flow diagram of search strategy and results.

An overview of the study search strategy and results is summarized in Figure 1.

### Decision Aid Appraisal

Of the 7 decision aids identified, the 6 that were designed to support patient decision-making are summarized in Table 3. The decisions that these resources addressed were as follows:

**TABLE 3.** Appraisal of patient-directed decision aids designed to support decision-making by patients choosing kidney transplantation

Title (reference)	Purpose/overview	Decisional focus	IPDAS		PEMAT scores	
**Meets qualifying criteria**	**Quality score** ^*a*^	**Understand-ability**	**Action**- **ability**
My Transplant Coach^16^	Help patients and families learn about dialysis and kidney transplant	Living vs deceased donor	Yes	75%	95.8%	93.3%
Inform Me about Increased Risk Donors^17^	Help patients make informed treatment decisions about whether to accept or refuse a kidney from an increased viral risk donor	Consent to an increased viral risk donor	Yes	70%	95.6%	33.3%
Benefits and risks of a kidney transplant^18^	Provide general knowledge and risks and benefits of receiving a kidney transplant.	Living vs deceased donor Accept or decline a kidney offer	No	–	73.7%	13.3%
Kidney transplant decision aid^19^	Inform patients regarding their treatment options and outcomes, including pros and cons of accepting higher vs lower quality kidneys and increased infectious disease risk kidneys	Consent to an increased viral risk donor Decisions around kidney quality	Yes	61%	72.5%	28.9%
Search centers that transplant patients like you^20^	Help to select transplant centers	Choice of transplant center	No	–	56.3%	9.5%
Is a kidney transplant right for me?^21^	To help patients in deciding whether a kidney transplant is the right option for them	Readiness for transplant Choice of transplant center	No	–	51.8%	40.0%

a Percentage calculated as the sum of points given for each quality dimension (ranked between 4—strongly agree and 1—strongly disagree), divided by the maximum theoretical points available.

IPDAS, International Patient Decision Aids Standards; PEMAT, Patient Education Materials Assessment Tool.

1. Readiness for transplantation
2. Living versus deceased donor
3. Choice of transplant center
4. Consent to an increased viral risk donor
5. Accept or decline a kidney offer
6. Other decisions around the quality of the donor kidney.

Only 3 of 6 patient-directed decision aids met the IPDAS qualification criteria; none met the certification criteria. Reasons for not meeting qualification criteria were that the available treatment options were not fully described (ie, the risks and benefits of 1 option only were considered) or the resource did not describe what it is like to experience the consequences of the options. Of the 3 decision aids meeting the qualifying criteria, quality scores ranged from 61% to 75%. Quality criteria that were not met included “the development process included a needs assessment with patients/health professionals” and “the development process included a review by professionals not involved in producing the decision support intervention.” Tools tended to have been field tested with patients facing the decision but not with the practitioners who counsel them.

PEMAT scores indicated that 4 of 6 decision aids were understandable, whereas only 1 was actionable (ie, scores exceeded 70%). Mean understandability scores averaged across raters ranged from 51.8% to 95.8%, with an intraclass correlation coefficient of 0.879 between raters, indicating excellent agreement. Mean actionability scores averaged across raters ranged from 9.5% to 93.3%, with an intraclass correlation coefficient of 0.812 between raters.

Individual PEMAT items that scored poorly included “the material does not include information or content that distracts from its purpose” (50% of the 18 scores given for this item indicated agreement), “the material provides a summary” (39% agreement), and “the material uses visual aids whenever they could make content more easily understood” (56% agreement). In terms of actionability, resources scored poorly with respect to the items “the material identifies at least 1 action that the user can take” (56% agreement), “the material breaks down any action into manageable, explicit steps” (17% agreement), and “the material provides a tangible tool whenever it could help the user take action” (28% agreement).

The highest-rated patient-directed decision aid in terms of PEMAT understandability score was the My Transplant Coach interactive decision aid (PEMAT score for understandability 95.8%), followed by InformMe (PEMAT score for understandability 95.6%). Strengths of My Transplant Coach include its visual cues and aids, use of everyday language, and delivery in active voice. Tools for calculating personalized prognosis information are also incorporated into this decision aid. The strengths of InformMe are that it is clear in its purpose and presents material in short sections with a logical flow.

One decision aid was identified that was designed for clinician use. The National Forum of End-stage Renal Disease Networks’ Kidney Transplant Toolkit is written for dialysis clinic staff to enable them to guide patients as they prepare for a kidney transplant.^22^ It is a comprehensive document that largely contains informational material, but it does contain a decision aid for determining “transplant readiness.” Although there is not a PEMAT equivalent for assessing the communication efficacy of decision aids designed for health professionals, the strengths of this resource are that its purpose is clear and that it provides a chart outlining specific actions to take.

### Risk Calculator Appraisal

The 10 unique risk calculators identified are listed in Table 4. Most were designed for clinician use, although 3 explicitly included patients among their intended users. Half did not involve an explicit choice but instead calculated donor-related risks or recipient prognosis only. Of the half that were designed around a specific choice, these choices included the following:

**TABLE 4.** Appraisal of risk calculators for risk prediction in deceased donor kidney donation and transplantation

Title (references)	Purpose/overview	Donor or recipient focused	Outcome/output	Decisional focus	Target audience	HSUS score
iChoose Kidney^23^	To calculate a patient 1- and 3-y risk of death with different treatment options	Recipient characteristics only	Predicted survival	Deceased vs living donor	Patients	86%
KDPI calculator^24^	To calculate a KDPI score for a hypothetical or actual donor	Donor characteristics only	KDPI	None	Clinicians	84%
EPTS calculator^25^	To calculate an EPTS score for a hypothetical or actual candidate	Recipient characteristics only	EPTS	None	Clinicians	83%
Kidney Transplant Outcome Prediction^26^	To calculate posttransplant survival probability based on recipient and transplant characteristics	Donor and recipient characteristics	Predicted survival Graft failure	Decline vs accept	Clinicians and patients	83%
KDPI-EPTS survival benefit estimator^27^	To calculate the predicted 5-y survival if a candidate remains on the waiting list vs accepts a given donor kidney	Donor and recipient characteristics	Predicted survival	Decline vs accept	Clinicians and patients	73%
EPTS score^28^	To calculate an EPTS score for a hypothetical or actual candidate	Recipient characteristics only	EPTS	None	Clinicians	68%
KDPI calculator^29^	To calculate the KDPI/KDRI for a given donor	Donor characteristics only	KDPI	None	Clinicians	66%
NHSBT Kidney Risk Communication Tool^30^	To visualize and communicate possible outcomes from the point of listing for deceased donor kidney transplantation.	Donor and recipient characteristics	Waiting time, death on waiting list, predicted survival, and graft failure	Decline vs accept	Clinicians	62%
Johns Hopkins IRD Kidney Transplant Calculator^31^	To predict the chance of survival if a patient accepts vs declines an increased-risk donor	Donor and recipient characteristics	Predicted survival	Decline vs accept (increased viral risk donor)	Clinicians	60%
Risk Adjustment Models: Posttransplant Outcomes^32^	Predictive model (hazard ratio) of graft and patient survival based on donor and recipient factors	Donor and recipient characteristics	Predicted survival	None	Clinicians	50%

EPTS, estimated posttransplant survival; HSUS, Healthcare Systems Usability Scale; IRD, increased risk donor; KDPI, Kidney Donor Profile Index; KDRI, kidney donor risk index; NHBST, National Health Service Blood and Transplant.

1. Whether to decline or accept a given deceased donor kidney offer
2. Whether to seek a deceased versus living donor kidney transplant
3. Whether to accept a risk of blood-borne virus transmission or remain on the waiting list.

Mean HSUS scores averaged across raters ranged from 50% (indicating major usability concerns) to 86% (indicating good usability with potential for improvements), with a median score of 71%. The intraclass correlation coefficient between raters was 0.793, indicating good interrater reliability.

The highest-rated risk calculators by the 3 reviewers were as follows:

1. The Emory University iChoose Kidney risk calculator (HSUS score of 86%), which estimates 1- and 3-y survival based on recipient characteristics and choice of treatment modality (living versus deceased donor)^4,23^
2. The United States Organ Procurement and Transplant Network calculators for Kidney Donor Profile Index (KDPI) and Expected Posttransplant Survival (EPTS; HSUS scores of 84% and 84%)^24,25^
3. The European Kidney Transplant Outcome Prediction risk calculator (HSUS score of 83%), which predicts mortality and graft survival outcomes after transplantation from brain-dead donors.^26^

The strengths of the iChoose Kidney risk calculator were that the information on the screen was easy to understand, assisted by well-designed visual aids that incorporate Gestalt principles of visual perception and easy-to-follow information hierarchies. Data entry processes were straightforward and results were reported as both numerical and percentage risks, supported with clear written interpretations (eg, “You are about 3 times more likely to die on dialysis than die with a kidney transplant in the next year”).

The strengths of the Organ Procurement and Transplant Network calculators for KDPI and EPTS included a clear layout, ease of data entry and navigation, as well as the provision of a written interpretation of the numerical score, which explained its correct and contextual interpretation. The strengths of the KTOP risk calculator were similarly clear layout and ease of data entry and navigation. It also provides a simple infographic for communicating risk estimates, combined with written interpretations of those risk estimates.

In terms of individual HSUS items, the lowest scores overall were in relation to the items “I understand how the tool creates its recommendations and scores” (mean score 3.1/5) and “On the screen I can find specific information I need quickly” (mean score 3.2/5). Information on the statistical models or methodology underlying risk scores or prognostic information was often absent or was otherwise difficult to locate, external to the tool, or difficult to follow. Common concerns included the following:

1. A lack of informative headings explaining the intent of the tool
2. Lack of context on what the calculator is for or how it should be used
3. Lack of a logical flow in how to enter information and/or poor general navigation
4. Charts that are difficult to interpret
5. No explanatory text to aid the interpretation of results in the correct context
6. Key results are not made prominent (eg, small font, hidden at the bottom of the page, no visual emphasis)
7. Undefined acronyms, use of jargon, undefined medical terms
8. Overly busy layouts and/or too much information on 1 page
9. No clear information hierarchy (eg, lack of headings, all font the same size)
10. Important information hidden behind tabs or external links
11. No (or very limited) information on data sources and/or methods.

### Gaps in Decision Support Resources

Table 5 maps the decision support tools that were identified by this environmental scan against the decisional needs of clinicians and patients on the pathway to deceased donor kidney transplantation.

**TABLE 5.** Decision support tools identified, mapped against the decisional needs of clinicians and patients with respect to deceased donor kidney transplantation

Perspective	Decision point	Decision support tool identified	Quality assessment^*a*^	References
Clinicians	Is my patient ready for kidney transplantation?	Decision aid	NA^*b*^	^16^
	Is this donor medically suitable for kidney donation?	None	.	.
	Does the biovigilance risk associated with this donor preclude acceptance for my patient?	Risk calculator	Medium	^31^
	Does this kidney offer a sufficient benefit to my patient?	Risk calculator	High	^26,27,30,32^
	Can my patient afford to wait for a better offer?	Risk calculator	High	^27^
Patients	Am I ready for kidney transplantation?	Decision aid	Low	^21^
	Should I pursue living or deceased donor kidney transplantation?	Decision aid and risk calculator	High	^16,18,23^
	Which transplant center should I list at?	Decision aid	Medium	^20,21^
	Should I consent to receiving offers of kidneys where there is some degree of biovigilance risk?	Decision aid	High	^17,19^
	Should I consent to receiving offers of marginal/expanded criteria donor kidneys?	None	–	–
	Should I accept or decline this kidney offer?	Risk calculator	High	^27^

a Low quality = highest PEMAT/HSUS score ≤50; medium quality = highest PEMAT/HSUS score >50 and <70; high-quality = highest PEMAT/HSUS score ≥70.

b Assessment tool not available.

HSUS, Healthcare Systems Usability Scale; PEMAT, Patient Education Materials Assessment Tool.

Most of the clinician-directed decision support tools that we identified were in the form of risk calculators. Our search strategy did not find any freely available tools that support clinician decision-making about whether a potential deceased donor is medically suitable for kidney donation to any recipient. We also did not find high-quality decision support tools that weighed the biovigilance risk associated with a specific potential donor against the needs of a matched potential recipient.

Most of the patient-directed decision support tools that we identified were in the form of decision aids. High-quality decision aids were identified that addressed (1) living versus deceased donor kidney transplant and (2) consent to accepting offers of kidneys from donors with an increased risk of infectious disease transmission. However, there was an identified gap in patient decision aids that support decision-making about whether to accept a specific kidney offer, particularly when the kidney in question was from a marginal donor. None of the tools identified framed this decision in terms of the expected time to the next offer or whether a better offer is likely. Instead, the decision was framed in terms of expected survival if transplanted with this kidney versus remaining on the waiting list.

## DISCUSSION

A range of freely available decision support tools for use in kidney transplantation was identified by our environmental scan, although usability, understandability, and actionability scores varied widely. Half of the patient-directed decision aids met the IPDAS qualification criteria and all were at high risk of bias, most commonly due to the lack of citations of evidence, information about when the tool was created and updated, or uncertainty estimates around prognostic information.^12^ Gaps in patient-directed resources included tools to support the decision to accept or decline a specific kidney offer. From the clinician perspective, we did not locate high-quality tools that would support decision-making on donor medical suitability or biovigilance risk from kidney donors.

Key concerns with regard to the communication efficacy of patient-directed decision aids included information overload, minimal use of visual aids or cues, and a lack of reinforcement of key points. Most patient-directed decision aids also did not clearly identify an action that the user could take or break that action down into steps. Developers of future decision aids should consider the PEMAT Checklist for Actionability when designing resources.^33^ Key concerns with regard to the usability of risk calculators were a lack of context on what the calculator was for or how it should be used, poor user experience design, charts that were difficult to interpret, and a lack of explanatory text to aid interpretation. Frequently, no information on data sources and/or methods was supplied, affecting face validity and the ability to correctly interpret and apply the results.

The availability of decision support tools that meet appropriate health literacy and usability standards is important given (1) the need for tools that can assist with the complexity of decision-making related to deceased donor kidney transplantation and (2) the recognized importance of shared decision-making in this space, where key discussion points may occur once only and with major consequences for the patient.^2^ Previous qualitative research into the decision support needs of patients on the kidney transplant waiting list found that misperceptions regarding the risks associated with different treatment options resulted in overestimation of the risks of transplantation, especially when the KDPI was high or there was an increased infection risk.^3^ These knowledge gaps persisted despite the provision of high-quality educational material, which led researchers to examine the emotional barriers to processing such information. Fear and overwhelm were identified as common emotions experienced by patients with kidney failure, which have been demonstrated to cause patients to rely on subconscious or implicit decision-making processes. This can result in a bias toward negative outcomes (eg, death) when evaluating prospective transplant options. The authors emphasized the need for individualized risk information and shared approaches to decision-making to overcome these challenges.^3^ A recent review of the values, preferences, and risk tolerance of people waitlisted for kidney transplantation similarly emphasized the importance of empathetic communication, transparent discussion of risks based on personalized prognostic information, and time for concerns to be heard and discussed.^34^

The lack of decision aids to support the decision to accept or decline a specific kidney offer is particularly noteworthy, given the evidence on outcomes of declined offers. Data from the United States show that patients who decline a kidney due to infection risk will typically wait longer to ultimately receive a lower-quality kidney, whereas accepting an increased-risk kidney was associated with a long-term survival benefit.^35^ A UK registry study found that recipients of previously declined kidneys experienced no significant difference in 5-y graft survival, compared with patients who were transplanted after declining an offer due to donor or organ-related reasons.^36^ Declining a kidney due to negative bias or inaccurate risk perception is a lost opportunity with potentially life-shortening consequences.

Decision support tools have been demonstrated to be an effective means of increasing knowledge about treatment options and outcomes, and of conveying more accurate perceptions of risks and prognosis.^6,37^ They also offer a structured way of describing the health problem and decision problem, evaluating options, and conceptualizing outcomes in the context of personal values and priorities.^1,6^ By making the relevant decisions salient in this manner, decision support tools accurately represent the unique trade-offs relevant to the patient, enabling reasoning competencies to increase while also reducing bias.^1^ A recent Cochrane review confirms the efficacy of decision aids in increasing the degree of congruence between patient values and care choices, reducing decisional conflict, and increasing patients’ perception of involvement in decision-making.^6^ Randomized controlled trial data comparing patient decision aids to usual care in cardiovascular, mental health, cancer, and joint replacement settings indicate that, when patients use decision aids, they experience increases in knowledge, are clearer about what matters most to them, and participate more in decision-making.^6^

Previous studies have also shown decision support tools to improve knowledge outcomes specifically in the setting of kidney transplantation.^38,39^ In a review of 27 decision aids to support shared decision-making in advanced kidney disease, Engels et al^38^ found that—of those decision aids that were evaluated for their effects on the outcomes of intended users—there was evidence of improved knowledge of treatment options, better decisional quality, and greater patient activation. This supports the findings of Irish et al^39^ who, in a systematic review of peer-reviewed decision support tools for people facing solid organ transplant, similarly found decision support tools increased knowledge outcomes compared with standard education controls. However, the degree to which conclusions can be drawn regarding their efficacy in actually promoting shared decision-making in this context is limited because of infrequent assessment of this as an outcome.^38,39^ Engels et al^38^ also found that only a minority of the tools were actually implemented in practice. Bekker et al^1^ have highlighted that decision support tools need to be integrated into existing treatment pathways and may require training for healthcare providers to deliver these tools in practice. Other studies have also additionally reported time constraints, disagreement on content or format, kidney failure–related cognitive decline, and organ scarcity as barriers to implementation.^37,40^ Our environmental scan and appraisal of such tools highlight some of the communication and usability concerns that are likely to be additional barriers to uptake.

By describing the usability and communication efficacy of decision support tools designed for use in kidney transplantation relative to validated usability scales^7^ and health literacy guidelines,^8^ our environmental scan points to the priorities for the development of future, better-quality, tools. A strength of our study is the use of the IPDAS, PEMAT, and novel HSUS instruments to appraise the usability and communication efficacy of these resources, highlighting key design considerations for future developers. Our study is limited in that we have only appraised freely available web-based tools accessible through a Google search, which are available in English. Undoubtedly, other relevant tools exist that are not publicly or freely available, not in English, or are regionally specific applications. Our sample of tools from multiple countries reflects heterogenous systems and sources of decisional conflict related to kidney transplantation; therefore, a tool designed for one setting may not be useful in another. Nonetheless, our findings on the strengths and weaknesses of publicly available resources from a design and communications perspective are still instructive for future developers. More knowledge-sharing with respect to best practice design would reduce duplication of effort and potentially lead to more rapid improvements in patient experiences and outcomes.

## Supplementary Material


