# Supplementary material for: Decision Support Tools in Deceased Donor Kidney Transplantation: An Environmental Scan and Appraisal of Online Resources
Source: Transplantation. 2025 Jul 14;109(12):e682–90. doi: 10.1097/TP.0000000000005473 (PMC12637122; doi:10.1097/TP.0000000000005473)
Supplement: Supplementary file 1 [file tpa-109-e682-s001.pdf]

Table S1

Adapted Healthcare System Usability Scale

Source: Ghorayeb A, Darbyshire JL, Wronikowska MW, *et al* Design and validation of a new Healthcare Systems Usability Scale (HSUS) for clinical decision support systems: a mixed-methods approach. *BMJ Open* 2023; 13: e065323 doi: 10.1136/bmjopen-2022-065323

Item	1=Strongly Disagree (crucial usability concern)	2=Disagree (major usability concern	3=Neutral (minor usability concern)	4=Agree (usability could be improved)	5=Strongly Agree (no usability concerns)
I found the information provided on the screen understandable					
I found it easy to navigate through the tool					
I will easily remember how to use the tool					
The screen layout makes it easy to see each piece of information					
On the screen, I can find specific information I need quickly					
I understand how the tool creates its recommendations or scores					
It is easy to correct a data entry error					
The tool highlights potential data entry errors					
The tool supports decision-making rather than dictating it					
